# Comparison of volatile chemical components of *Cystoseira crinita* Duby by hydro-distillation (HD) and solid-phase microextraction (SPME) methods and antimicrobial and scolicidal activities of essential oil and extracts

**DOI:** 10.55730/1300-0527.3314

**Published:** 2021-11-25

**Authors:** Tayyibe Beyza YÜCEL

**Affiliations:** Giresun University, Health Services Vocational School, Giresun, Turkey

**Keywords:** *Cystoseira crinita* Duby, volatile compound, antimicrobial effect, scolicidal effect, lethal dose, probit analysis

## Abstract

This study aims to derive an essential oil from *Cystoseira crinita* Duby, analyze the chemical composition of the essential oil, discover the antimicrobial activities of the oil and the extracts, and investigate the scolicidal activities of the extracts. The volatile organic compounds of *Cystoseira crinita* Duby were determined by GC/MS-FID using both hydro-distillation (HD) and solid-phase microextraction (SPME) methods. As a result of the essential oil analysis, 97.14% of 17 compounds and 93.13% of 19 compounds were elucidated. The main compounds identified were hexanal (31.802%), n-hexadecanoic acid (12.654%), trans-β-ionone (9.118%), 2*E*-hexenal (15.955%), heptadecane (15.729%) and tetradecane (13.458%). In addition, hexane, dichloromethane, chloroform, and methanol extracts of the algae sample were prepared. The antimicrobial activities of the essential oil and extracts on gram-positive bacteria, gram-negative bacteria, and fungi microorganisms were studied. The results revealed activity within the zone diameter range of 8–16 mm. It was observed that all extracts and the essential oil itself showed high activity against the *Pseudomonas aeruginosa* ATCC 27853 microorganism. The chloroform and dichloromethane extracts were also found to demonstrate a high level of efficacy against *Bacillus cereus* ATCC 10876. Furthermore, viability detection was performed and the scolicidal effects of the extracts on protoscoleces were assessed. All extracts showed a strong scolicidal effect at the dose of 15000 μg/mL. For each solution, the difference between hours at each dose and the difference between doses at every hour were compared by One-Way ANOVA. The values of lethal concentration doses (LD_50_ and LD_90_) were calculated using Probit Analysis. This study provides information about the effects of *Cystoseira crinita* Duby algae extracts and suggests that the experimental studies needed for their use in live cells should be performed.

## 1. Introduction

Microalgae and macroalgae are vegetative organisms. Macroalgae are aquatic organisms and remarkably dominant in marine ecosystems. They are also widely consumed as food in coastal regions, especially in the Far Eastern countries such as China, Korea, and Japan. Microalgae are classified as brown (Phaeophyta), red (Rhodophyta), and green (Chlorophyta) depending on their pigmentation and if they bear a flagellum [1,2]. Approximately 9000 species have been recorded worldwide, while there are approximately 5000 species recorded and 600 bibliography and distribution records in Turkey [3,4].

Since algae are photosynthetic organisms, they can provide seawater with oxygen (O_2_) while also constituting a reliant food source for marine organisms such as shrimp and jellyfish with the acids, alkaloids, amines, cellulose, enzymes, glycosides, trace elements, inorganic minerals, lipids, sterols, ceroids, vitamins, amino acids, proteins, pepsins, and volatile compounds [2,5,6]. Algae’s bioactive components (secondary metabolites, diterpenes, brominated sesquiterpenes, sesquiterpenes, polyphenols, flavonoids, minerals, polysaccharides, polyunsaturated fatty acids, vitamins, etc.) means they are often used for their sedative, muscle relaxant, and edema relieving properties in both medicine and pharmacology. They are also used as an agricultural fertilizer and in the production of polysaccharides, such as agar and alginate, in the food industry. Additionally, algae can be used to produce biofuels such as ethanol, butanol, and biogas [7–9]. Many studies show that algae form resistance to bacteria, fungi, and viral pathogens [10,11]. In addition, red and green algae appear to have a preventative effect on the skin, breast, and intestinal cancer [2].

There are more than 7000 species of brown algae, yet less than one percent are found in freshwater environments, while the remaining part can be found in the seas. Some species are microscopic, and some can reach up to 30 meters [12]. Brown algae are also rich in polyphenols, which have antioxidant properties. They are used in the rubber industry, dyes, ice cream, and plastic freezers due to their alginate content [13,14]. *Fucus vesiculosus* (Bladderwrack), also known as kelp, is part of the brown algae class, and it contains a high amount of iodine. It is known to have a high antioxidant and antiinflammatory effect and can be used to treat hormonal diseases.

Essential oils derived from plants are aromatic mixtures obtained from root, stem, leaf, fruit, shell, and flower. They are liquid at room temperature and can crystallize with ease [15]. The following is a list of academic studies regarding the essential oil analyses of algae and their anticancer [16], antileukemic [17], antioxidant [18,19], antimicrobial [20], antifeedant [21], antifouling [22], antimalarial [23], and cytotoxic [24,25] properties.

Due to the algae’s popularity in far eastern countries as a food group for its cosmetic and medicinal uses and its wide range of biological activity, there are many studies worldwide detailing its benefits. But the number of such studies is quite limited in Turkey, and what studies, primarily using algae collected from the Aegean Sea, were conducted. Therefore, this study was carried out in the Black Sea due to the small number of such studies in this region.

There are a few reports on the antibacterial, antiprotozoal, antimycobacterial, and cytotoxic activity of methanol extract of *Cystoseira crinita* Duby grown in different parts of the world including Turkey. In these studies, biological activities were found quite high. Since this species has been found to have wide biological activity in the limited number of studies in the literature, it is aimed to determine the chemical composition and different biological activity studies of this species.

This study aims to detect the volatile components of *Cystoseira crinita* Duby, which is in the brown algae class, situated around the Sinop province, using both hydro-distillation (HD) and solid-phase microextraction (SPME) methods, and to extract Soxhlet using chloroform (Ch), dichloromethane (DCM), hexane (H) and methanol (MeOH), to determine the antimicrobial activities of both essential oil (HD) and extracts and the scolicidal effects of extracts. There are already studies that determine the antimicrobial and antioxidant properties of various extracts of *C. crinita* collected from different seas worldwide, including Turkey [26,27]. However, no studies have been found regarding the antimicrobial analysis of essential oil and solvent extracts except for methanol and chloroform of *C. crinita* Duby. Also, in previous studies, there is no scolicidal activity of solvent extracts. Although there are antimicrobial activity studies, this study differs in terms of the essential oil composition and test-microorganisms used for antimicrobial activity.

## 2. Materials and Methods

### 2.1. Algae material

*C. crinita* was collected in November 2017 from Sinop, Akliman and Merkez Tersane provinces. The algae were stored in polyethylene bottles and transported to the laboratory using a cold chain. The sample was first rinsed with seawater to remove sand particles, then cleansed of any remaining sea organisms. It was then left to dry in the open air at room temperature under shade. The dried samples were then cut into smaller sections using a mixer in the laboratory and prepared for testing.

### 2.2. Isolation of essential oil

The essential oil was extracted using a Clevenger apparatus. To achieve this, the dried and crumbled algae sample *C. crinita* (65 g) was weighed and placed into a two-liter flask and then mixed with distilled water. The essential oils were extracted using a Clevenger apparatus connected to the cooling bath (−12 °C) over four hours. The derived essential oils were extracted from the Clevenger apparatus using HPLC purity *n*-hexane (0.5 mL) and transferred into brown vials. The amount of oil obtained as a result of hydro-distillation with a Clevenger apparatus was weighed and found to be 15.5 mg, respectively. It was then weighed and stored at +4 °C while awaiting the antimicrobial activity tests.

### 2.3. SPME analysis

Solid phase micro-distillation was used for this process. One gram of air-dried algae material was grounded and placed into a vial for SPME analysis. Vials were 10 mL in volume and sealed with a silicone-rubber septum cap. A polydimethylsiloxane/divinylbenzene fiber was used to extract the volatile components. Extractions were achieved with magnetic stirring.

The fiber coating fibers were conditioned for five minutes at 250 °C in GC injector before being placed into the headspace. Temperature, incubation, and extraction times were set according to the experiment. SPME was processed at 50 °C with an incubation time of 5 min and an extraction time of 10 min. Each sample was then analyzed and reported [28].

### 2.4. Extraction using the Soxhlet apparatus

Thirty grams of dried *C. crinita* portions were weighed out, put into Soxhlet cartridges, and placed in the reflux. The soxhlet extraction took place over 24 h by adding 250 mL of chloroform (Ch), dichloromethane (DCM), hexane (H), and methanol (MeOH) into each flask. The end solution was then passed through the evaporator and dried. The remaining residue was weighed (3.2%, 2.5%, 2.5%, and 3.8%, respectively) and stored at +4°C awaiting biological activity tests. All solvents used were 95%–98% pure and Sigma–Aldirch branded.

### 2.5. Gas chromatography-mass spectrometry (GC-MS/FID)

The gas chromatography chromatography-flame ionization detector (GC-FID) analysis was carried out on a Shimadzu QP2010 plus gas chromatography coupled to a Shimadzu QP2010 ultra mass selective detector. The fiber-containing, extracted aroma compounds were injected into the GC-MS injector (split mode). Separation took place with a Restek Rxi-5MS capillary column of 30 m length, 0.25 mm i.d., and 0.25 μm phase thickness. The oven program began at an initial temperature of 60 °C for 2 min, which was then increased to 240 °C for three minutes, and then a final temperature of 250 °C for four minutes. The injector temperature was 280 °C and the split ratio was 1:20. The carrier gas was helium (99.999%), with a constant flow rate of 1 mL/min; sample size was 1 μL. Detection was processed in electronic impact mode (EI); ionization voltage was fixed to 70 eV, and scan mode (40–450 m/z) was used for mass acquisition.

### 2.6. Analysis of components

The retention indices (RI) of the components were determined with n-alkanes (C_6_-C_30_) using the Koyats method. Comparison retention indices of volatile components (relative to C_6_-C_30_ alkane standards) were identified by comparing the mass spectra of the two libraries (FFNSC1.2 and W9N11).

### 2.7. Antimicrobial Analysis

Antimicrobial activity was performed using the disc diffusion method as described by Demirel et al. [29]. For antimicrobial activity, a total of 19 bacterial species were used. The gram-positive bacteria were *Bacillus (B). subtilis* ATCC 6633, *Bacillus cereus* ATCC 10876, *Bacillus megaterium* DSM32, *Staphylococcus (S.) aureus* ATCC 29213, *Staphylococcus aureus* ATCC 25923, *Enterococcus faecalis(E.)* ATCC 29212, Metisillin Resistant *Staphylococcus aureus* (MRSA) ATCC 67101, *Staphylococcus epidermidis* ATCC 12228; and the gram-negative bacteria were *Cedecea(C.) neteri ATCC 33855, Escherichia (E.) coli* ATCC 25922, *Escherichia coli* ATCC 36218, *Pseudomonas (P.) aeruginosa* ATCC 27853, *Pseudomonas aeruginosa* ATCC 9027, *Klebsiella (K.) pneumoniae* ATCC 13883, *Acinetobacter (A.) baumannii* ATCC BAA-747, *Enterobacter (En.) aerogenes* ATCC 13048, *Citrobacter (Cb.) freundii* ATCC 43864, *Salmonella (Sa.) typhimurium* ATCC 14028, *Proteus (Pr.) mirabilis* ATCC 43071. The *Candida (Ca.) albicans* ATCC 10231 strain was used for antifungal activity.

To determine the antimicrobial activity of *C. crinata* essential oil, the essential oil was dissolved in DMSO at a concentration of 8.61 mg/mL. Similarly, in antimicrobial studies of Ch, MeOH, H, and DCM extracts of the algae sample, the extracts were dissolved in DMSO with a concentration of 15 mg/mL. Six mm sterile blank discs were soaked in 2 different amounts of extracts each prepared using different solvents, A:15 μL and B:30 μL. To measure the essential oil and extracts’ antimicrobial effect, the microorganisms tested were inoculated in a Müller Hinton Broth and the yeasts in a Sabouraud Dextrose Broth, which were incubated for 18 h at 37 °C. Turbidity measurements were then adjusted with a densitometer to McFarland No: 0.5. Each broth was adjusted to McFarland No: 0.5 under sterile conditions. From those, 100 μL was put into all Mueller Hinton Agar Broths and equally distributed to Petri dishes with a sterile swab. The prepared discs were placed into Petri dishes, and the microorganisms were inoculated. After 24 h of incubating at 37°C, the discs’ zone diameters and measured and the values were noted in millimeters. Tobramycin (Bioanalyse, 10 μg/disc), Nystatin (Bioanalyse, 30 μg/disc), and Vancomycin (Bioanalyse, 30 μg/disc) standard antibiotic discs were used as controls.

### 2.8. Scolicidal analysis

The viability of the protoscolices obtained from a cow liver cyst hydatid was determined using a 0.1% eosin solution. Among cyst hydatid treatment methods, percutaneous interventions can be applied according to its localization. Scolicidal agents can be used in these interventions [30]. Scolicidal agents include 20% or 30% hypertonic saline, 95% ethanol, 0.5% cetrimide. Researchers have stated that percutaneous interventions are safe and effective [31,32]. The solution was dissolved in sterile saline. Its final concentration was adjusted to 15000 μg/mL and was homogenized and sterilized by filtering the membrane.

Sterile saline of 100 μL was added to the wells of the sterile microplate. A total of 100 μL of *C. crinita* extracts were then added to the first wells and diluted to a 1/2 ratio. Once diluted, a 3500 piece/mL concentration of 100 μL protoscolices was added to the wells. As a control, only 100 μL protoscolices with a certain dilution were added to wells 9 and 10. Counting was used to determine the viability rates of protoscolices at certain times. Each counting was performed twice, and the results were averaged. A frequently used in the study routine NaCl solution (30%) was included as a scolicidal agent.

### 2.9. Statistical analysis

The descriptive statistics of the data set are expressed as a mean ± standard deviation (SD). The differences between means were compared by the one-way ANOVA followed by Tukey’s post-hoc test. Lethal dose values (LD_50_ and LD_90_) were determined using probit analysis at certain times. A p-value was considered statistically significant at ≤0.05. All statistical analyses were performed using the SPSS v26 (IBM Inc., Chicago, IL, USA) and the Minitab 19 (Minitab Inc., State College, PA, USA) statistical software.

### 2.10. Findings

The amount of essential oil extracted from the steam distillation of *C. crinita* weighed to be 15.5 mg (0.085% w/w). A total of 17 and 13 compounds, comprising 97.14% and 93.13% of the HD and SPME, respectively, were identified (Table 1). Weighted components in the essential oil derived by hydro-distillation were 38.88% aldehyde, 18.68% terpenoid class compounds, while the SPME main component class was determined to be 53.89% aldehyde and 36.73% hydrocarbons compounds (Table 1). Compounds identical to the SPME and HD analyses were found to be hexenal (31.80% and 12.19%), (2*E*,4*E*)-heptadienal (6.50% and 5.95%), pentadecane (1.55%–1.35%), and hexadecane (3.02%–4.73%). In this context, a difference in content can be observed. Main components were hexenal (31.80%), *n*-hexadecanoic acid (12.65%), trans-β-ionone (9.11%) in HD analysis and 2*E*-hexenal (15.95%), heptadecane (15.72%) and tetradecane (13.45%) in SPME analysis.

**Figure 1:**
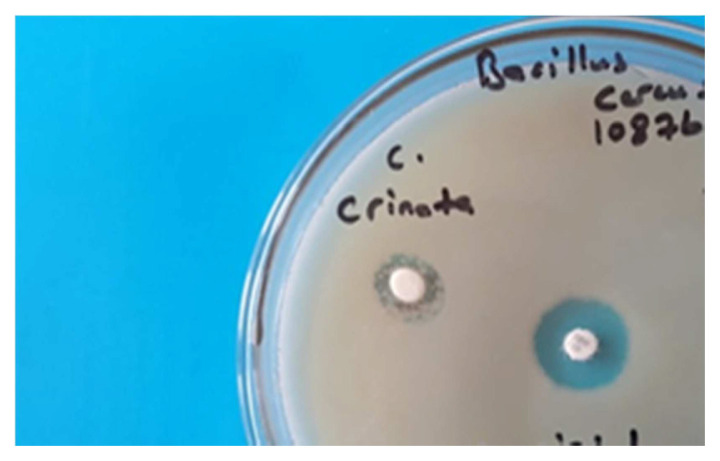
Antimicrobial test view of essential oil on *B. cereus* A:15 μL, B:30 μL, Tob: Tobramycin, Nys: Nystatin.

The antimicrobial activity results of the essential oil obtained by HD were measured on five microorganisms, including *S. aureus* 25923, *MRSA, B. cereus* ATCC 10876, *P. aeruginosa* 27853, *E. coli 25922*, *Ca. albicans ATCC 10231*, diameter values were determined medium 14–13 mm zone on *B. cereus* and *P. aeruginosa* according to Tobramycin standard agent. But no antimicrobial activity was observed against the fungus *Ca. albicans* according to standart agent as Nystatin. The results are shown in Table 2. The Petri view of the essential oil zone on *B. cereus* can be seen in Figure 1.

The antimicrobial effect of extract’ *C. crinita* according to the disc diffusion method is displayed in Table 3. The antimicrobial activities of both Ch and DCM extracts of *C. crinita* at 30μL concentration weren’t observed to have an antimicrobial effect against *Pr. mirabilis*; Ch, H, and MeOH extract against *E. coli* ATCC 25922 and the H and Ch extract against *Sa. typhimurium* ATCC 14028. In the 8–16 mm zone range at both 15 μL and 30 μL concentrations, the antimicrobial effect was observed only against *B. cereus* ATCC 10876 and *P. aeruginosa* ATCC 27853. The highest zone values of all extracts were found against *P. aeruginosa* ATCC 27853 according to Tobramycin as standard agents. All extract concentrations except for 15 μL dose of M extract were determined antifungal activity against *Ca. albicans* ATCC 10231.

**Figure 2:**
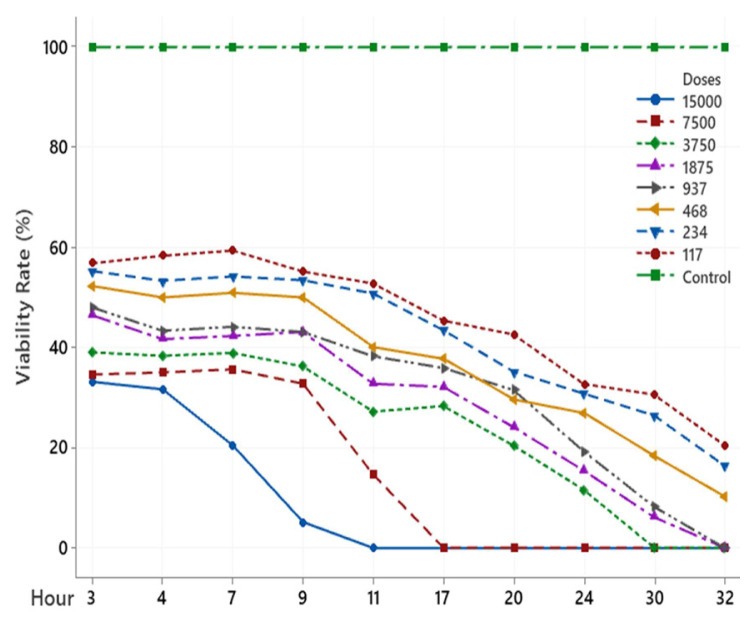
Ch extract (%) viability rate.

**Figure 3:**
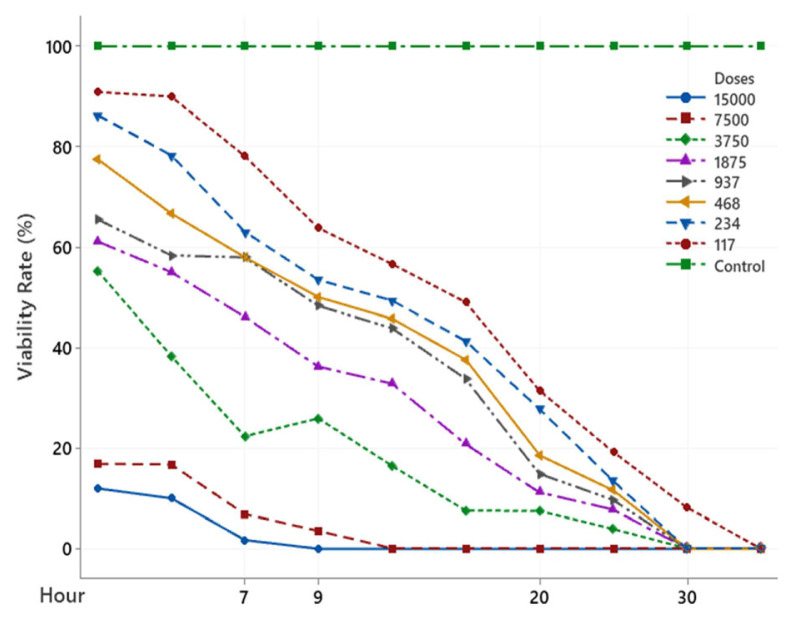
DCM extract (%) viability rate.

**Figure 4:**
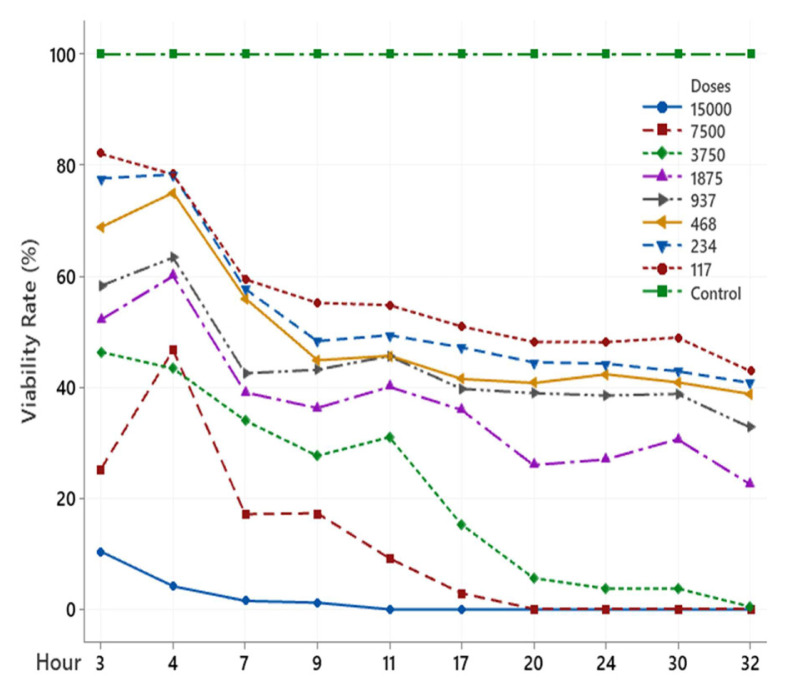
H extract (%) viability rate.

**Figure 5:**
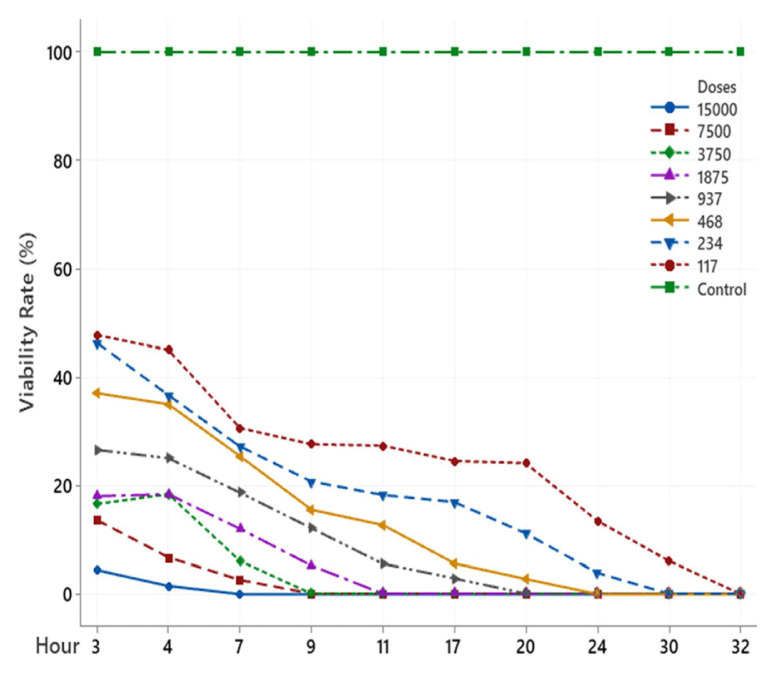
MeOH extract (%) viability rate.

**Figure 6:**
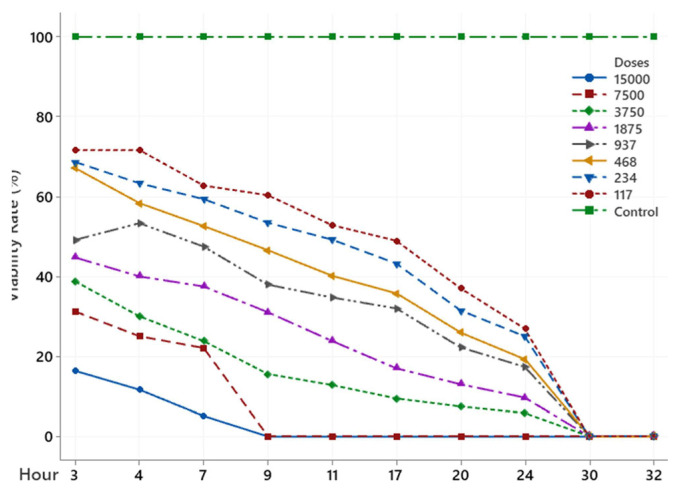
NaCl extract (%) viability rates.

The scolicidal effect of Ch, DCM, H and MeOH extracts of *C. crinita* prepared in different concentrations on protoscolices at different hours are shown in Figures 2–6.

When examining the graphs, the extracts of *C. crinita* prepared in different concentrations were seen to decrease with time except for the control group. When the applied dose effect was examined, the decrease in the viability rate per time was observed to be faster as the dose amount increased.

The scolicidal effect of the *C. crinita* extracts in organic solvents at different concentrations on protoscolices, according to the dose applied at different times, is shown in Table 4 to Table 8.

It was observed that the DCM extract prepared at different concentrations of *C. crinata* had a very strong scolocidal effect at 15000 μg/mL. It has been determined that it affects the parasites in a very short time compared to other concentrations, and no live parasites have been encountered, especially at the 9th h (Table 4).

When Table 5 was examined, it was found that the hexane extract of *C. crinata* had a very strong scolocidal effect at the concentration prepared at 15000 μg/mL. No live parasites were found at the 11th..

As other extracts DCM and Hexane, it has been determined that Chloroform extract has a very strong scolocidal effect at a concentration of 15000 μg/mL. No live parasites were found at the 11th. (Table 6).

It was determined that the methanol extract had a very high scolocidal effect on parasite cells. and no living parasites were found after the 7th hour (Table 7). It is seen that the most effective concentration is 15000 μg/mL as other extracts of *C. crinita*.

It was observed that the NaCl solution used as a scolocidal agent had a very strong scolocidal effect at a concentration of 7500 μg/mL, and no live parasites were found at the 9th. (Table 8).

Determining the scolicidal effect in terms of parasite viability, the difference between the exposure times and the doses of plant extracts was found to be statistically significant (p <0.05). Although this varies according to the extract doses, an interpretation can be made as the dose increases the parasite’s viability rate decreases. Again, a decrease was observed in the viability of the parasite, depending on the exposure time.

*C. crinita’s* 15000 μg/mL concentration showed a strong scolicidal effect in all of the Ch, DCM, H, and MeOH solvents. No living parasites were observed at the 7th h with the MeOH extract of *C. crinita* and at the 9th h with the DCM extract (Table 9–10).

From DCM and H extracts of *C. crinita*, the LD_50_ value of DCM observed in the protoscolices at the 9th h was determined to be 498.5 μg/mL, and its LD_90_ value was 6800.0 μg/mL; the LD_50_ value of H extract in protoscolices at 11th h was determined to be 368.2 μg/mL, and its LD_90_ value was 13675.4 μg/mL (Table 9).

The LD_50_ value of the *C. crinita* Ch extract observed in protoscolices at the 11th h was determined to be 270.4 μg/mL, and its LD_90_ value was 10436.6μg/mL; the LD_50_ value of the M extract observed in the protoscolices at the 7th h was determined to be 32.0 μg/mL, and its LD_90_value was 1863.2 μg/mL (Table 10).

The LD_50_ value observed at the 9^th^ hour of the NaCl solution used as a scolicidal agent in protoscolices was determined to be 370.0 μg/mL, and its LD_90_ value was 4331.7 μg/mL (Table 11).

## 3. Discussion

Essential oils have been used for various purposes over the years. The composition of essential oils is terpenic or non-terpenic volatile compounds. Compounds that make up essential oils are predominately hydrocarbons and oxygenated derivatives of hydrocarbons, but structures such as alcohol, carboxylic acid, ester, aldehyde, ketone, etc. can also be found [33,34].

Terpenes are formed by bonding with isoprene (C_5_H_10_) units. They enter into the essential oil structure in their monoterpene, sesquiterpene, diterpene, and their oxygenated forms. The terpenic compounds in the essential oil are thought to be the reason for their wide range of biological activities. That’s why the contents and biological activities of essential oils have become a focus of interest in scientific fields and why relevant studies have grown in importance [35–37].

They have grabbed many scientists’ attention due to their wide range of utilization in areas such as cosmetics, medicine, food industry, dentistry, oral care products, perfumery, dyeing, aromatherapy, and phytotherapy. For this reason, the chemical structures and biological activities of essential oils have become a subject of curiosity in recent years [38].

While examining scientific studies, it has been observed that the extraction process has been applied to either fresh or dried algae [39,40]. However, the extracts prepared with fresh samples were found to have higher biological activity than those found in studies with dried samples [41]. The commonly used drying processes used in studies were freeze-drying (lyophilizer) and open-air drying [42–44]. In this study, the algae samples were dried in the open air, under shade, for two to three days after being cleansed of foreign material such as epiphytes and sand.

The compound classes and amounts having the most considerable quantities in the structure of *C. crinita* were found respectively in HD and SPME to be aldehyde (38.88%, 53.89%), terpene and terpenoid compounds (18.684%, 2.52%), and hydrocarbons (10.63%, 36.73%). In the essential oil composition of carboxylic acid compounds, tetradecanoic acid (2.694) and hexadecanoic acid (12.654%) were detected, and the ratio of carboxylic acid compounds in the total essential oil was determined to be 15.35%. Discovered in the essential oil structure derived with HD were the ketone (11.83%) and alcohol (2.24%) class compound structures. The compounds determined in the essential oil composition were collected under a total of 6 classes. Using SPME, volatile components were grouped under three classes: aldehyde, hydrocarbon, and terpene. When examining the effect of the essential oil on a total of five microorganisms, it was observed a high zone formation (14 mm) against gram-positive bacterium *B. cereus* ATCC 10876, and a high zone (13 mm) against the gram-negative bacterium *P. aeruginosa* ATCC 27853. The tobramycin zone value as a standard antibiotic disc was observed to be 18 mm for *B. cereus* and 20 mm for *P. aeruginosa*.

We continued to study the antimicrobial activities of the *C. crinita* extracts in organic solvents. Solutions of 15 and 30 μL for each of the extracts were prepared. Extracts were found to be antimicrobial effective on 8 gram-positive, 11 gram-negative, one fungus, for a total of 20 microorganisms. From the two different concentrations prepared, the extracts with a concentration of 30 μL were observed to form a more effective zone diameter compared to the 15 μL extracts. Extracts with a concentration of 15 μL displayed effects predominately on gram-positive bacteria among 20 microorganisms (*S. aureus* ATCC 29213, MRSA ATCC 67101, *B. cereus* ATCC 10876). Extracts with a concentration of 30 μL generally displayed an effect on all microorganisms in the zone range of 6.5–16 mm except for *Pr. mirabilis* and *E. coli* ATCC 25922. In the extracts with both the 15 and 30 μL concentrations, antimicrobial effects were only observed on *B. cereus* and *P. aeruginosa*.

Plant extracts are highly complex mixtures containing many components. If the disc diffusion method used in this study is indicated by including standard drugs, it can be evaluated. However, the ineffective molecules should be restudied later with dilution methods to eliminate the diffusion problem in the agar method.

Kamenarska et al. (2002) obtained different compounds from the present study in the GC/MS analysis of *C. crinita*, collected from the Aegean Sea at the coast of Kaş. However, the terpene class compounds found by the researchers were similar. This may have been caused by shifting climates and the natural passing of time [26].

Ibtissam et al. (2009), in their study on 32 algae including *C. crinita* in Morocco, the algae samples they examined obtained methanol extracts using the Soxhlet extraction method and examined the antibacterial activity of the extract they obtained. In the study, a zone higher than 20 mm was detected on *S. aureus* ATCC 25923, but it didn’t affect other microorganisms [27]. It was observed that the value on *S. aureus* was higher than the results as in our case, which can be explained by the environment, locality, and the subspecies of the algae used.

When examining the study, Mhadhebia et al. [45] use various algae extracts prepared with Ch and M to determine the antibacterial activity, including *C. crinita*. The values for the Ch extract of *C. crinita* against *S. aureus* ATCC 25923 and *E. coli* ATCC 35218 in the 6–9 mm zone range are parallel to the values found in our study. However, the antimicrobial values of the M extract were observed to be different.

Selçuk et al. [46] performed different biological activity tests, such as the antiprotozoal, antimycobacterial, and cytotoxic activity for M extracts of algae samples including *C. crinita* and found relatively high values.

In another study, Berber et al. *C. crinita* Duby and *U. intestinalis* seaweed collected from the Sinop coast dried in the open air and coast and prepared methanolic extracts mixing the algae samples in methanol in a water bath for 24 h. They studied the antioxidant and antimicrobial activities of the obtained extracts. Antimicrobial activity was observed against *S. aureus ATCC 25923* and *S. epidermidis* range of 17–20 mm inhibition zone, while smaller zone diameter was found in our study. The same value antimicrobial activity was found against *B. subtilis* and *E. faecalis* 9–10 mm zone. Although, no antimicrobial activity against *E. coli* and *Ca. albicans* was found in [47], we found.

In this study, due to the components having biological activity in the *C. crinita* structure, it was thought that it is likely to display scolicidal effects along with the antimicrobial activity. Results from the scolicidal effect study of the algae sample extracts in four organic solvents (Ch, DCM, H and M), was observed to decrease over time except for the control group. Additionally, the extract dose prepared at a concentration of 15,000 μg/mL was observed to have a strong scolicidal effect.

According to the reference information reached in different studies, a scolicidal agent, formol, hypertonic glucose solution, alcohol, hypertonic NaCl (3%;10;20), chlorine hexidine, cetrimide, AgNO_3_, povidone-iodine, alcohol, 3% H_2_O_2_, albendazole solution, iodine were used [48–50]. Among the scolicidal agents used, formol, hypertonic saline, and alcohol were found to have the highest risk [51, 52]. In this study, the effect of Ch, DCM, H, and M extracts of *C. crinita* on protoscolices was investigated in parallel using NaCl as a scolicidal agent.

Ozcelik et al. [53] reported that *Allium sativum* was effective on protoscolices. Again, Ozcelik et al. [54] found that propolis also affected daughter vesicles within 10 min. In the study, the M extract of *C. crinita* showed no live parasites at the 7th h. Additionally, the DCM extract showed no parasites at the 9th h. No viable protoscolices were observed at the 11th h for the Ch and H extracts. When the application doses were compared to the Tukey test, application dose rates showed a significant change according to the doses (p < 0.05). In the study, LD_50_ and LD_90_ values were also examined by probit analysis. Accordingly, the LD_50_ value observed in the protoscolices at the 9th h of DCM extract of *C. crinita* was determined to be 498.5 μg/mL; LD_90_ value of H extract was determined to be 6800.0μg/mL, LD_50_ value observed at the 11th h as 368.2 μg/mL; LD_90_ value was 13675.4μg/mL, the LD_50_value observed in protoscolices at the 11th h of Ch extract was 270.4 μg/mL; LD_90_ value was 10436.6; (please remove coma) μg/mL; LD_50_ value wa 32.0 μg/mL, and LD_90_ value was 1863.2 μg/mL at the 7th h of M extract. With this information in mind, it can be concluded that *C. crinita* extracts can be used as potential scolicidal agents.

## 4. Conclusion

The volatile components of *C*. *crinita* were observed with both HD and SPME in the GC-MS/FID device, and their structures were clarified in this study. Terpene and terpenoids, carboxylic acid, aldehyde class compounds, which were not identified in previous studies, were found to be present here. In the antimicrobial activity study of essential oils, *C. crinita* was observed to have a high activity level on our study’s microorganisms. Most of the extracts prepared with organic solvents were affected with a value of 6.5 to 16 mm zone diameter.

Furthermore, organic solvent extracts of *C. crinita* were determined to have an additional use as bactericidal and scolicidal agents. Thus, it was concluded that LD_90_ dose can be increased for a faster effect or decreased for a slower effect. For *C. crinita* to be used on living cells, it is recommended that controlled experiments are performed with experimental animals and that dosage levels are made to adjust the amount of antimicrobial effect.

## Figures and Tables

**Table 1:** Identified components in the essential oils of of *C. crinita*.

	Retention Time	Compounds	*HD* % Area^a^	SPME %Area^a^	Exp. RI^b^	Lit. RI
1	8.593	Hexenal	31.802	12.194	801	803
2	10.022	(*E*)-2-Hexenal	-	15.955	852	851
3	10.162	(*Z*)-3-Hexenol	1.558	-	858	858
4	11.710	Heptanal	-	12.481	-	903
5	14.724	1-octene-3-ol	0.679		977	978
6	15.305	Furan-2-pentyl	2.072		991	993
7	15.621	(*2E,4E*)-Heptadienal	6.507	5.957	1009	1012
8	17.022	Limonene	-	2.518	1031	1030
9	17.646	(*E*)-2-Octenal		1.087	1047	1049
10	18.135	Benzene acetaldehyde	0.579		1056	1052
11	20.055	Nonanal	-	3.880	1102	1101
12	24.406	Decanal		2.337	1202	1201
13	28.220	Tridecane^c^		1.459	1303	1300
14	32.203	Tetradecane^c^		13.458	1403	1400
15	34.408	Geranyl acetone	2.411		1450	1453
16	35.766	1-Pentadecene	3.980		1485	1489
17	35.898	Trans-β-ionone	9.118		1489	1487
18	36.040	Pentadecane	1.551	1.353	1502	1500
19	40.008	Hexadecane	3.024	4.731	1601	1600
20	41.215	Benzophenone	7.950		1625	1626
21	43.177	Heptadecane^c^	-	15.729	1701	1700
22	43.963	Pentadecanone	3.417		1715	1710
23	45.383	Tetradecanoic acid	2.694		1758	1763
24	48.017	Hexahydrofarnecyl acetone	4.579		1849	1848
25	51.778	n-Hexadecanoic acid	12.654		1960	1966
26	56.296	Phytol	2.572		2114	2110
		Total	%97.14	%93.13		

a RI calculated from retention times relative to that of n-alkanes (C_6_ -C_30_) on the non-polar HP-5 column.

b % Area obtained by FID peak-area normalisation.

c Identified by authentic samples.

**Table 2:** Antimicrobial activity of the essential oil of *C. crinite*.

Microorganisms	Inhibition Zone(mm)		Standards	
	A	B	Tob	Nys
*S. aureus* 25923	-	-	22	-
*MRSA*	-	-	20	-
*B. cereus ATCC 10876*	-	14	18	-
*P. aeruginosa* 27853	-	13	20	-
*E. coli* 25922	-	-	22	-
*Ca. albicans ATCC 10231*	-	-	-	24

**Table 3:** Antimicrobial effect of extracts of *C. crinita* on microorganisms.

Microorganisms	Inhibition Zone (mm)										
	Ch^a^		DCM^a^		H^a^		MeOH^a^		Standard^a^		
	A	B	A	B	A	B	A	B	Tob	Nys	Va
*S. aureus ATCC 25923*	6,5	9	6,5	9	-	6,5	7	8	20	-	-
*S. aureus ATCC 29213*	-	9	-	9	-	8	-	9	20	-	-
*S.epidermidisATCC 12228*	-	7	-	7	-	10	-	8	-	-	-
MRSA ATCC 67101	6,5	8	-	6,5	6,5	7	7	9	-	-	17
*B. cereus ATCC 10876*	8	12	8	12	6,5	7	6,5	7	22	-	-
*B. megaterium DSM32*	-	10	-	10	-	6,5	-	8	18	-	-
*B. subtilis ATCC 6633*	-	9	-	8	-	-	-	10	18	-	-
*E. faecalis ATCC 29212*	-	7	-	8	-	9	-	10	16	-	-
*P.aeruginosa ATCC 27853*	10	10	7	9	12	16	12	14	18	-	-
*P.aeruginosa ATCC 9027*	-	10	-	10	-	7	-	7	20	-	-
*E. coli ATCC 25922*	-	-	-	6,5	-	-	-	-	20		-
*E. coli ATCC 36218*	-	9	-	8	-	7	-	9	20	-	-
*K.pneumoniae ATCC 3883*	-	8	-	8	-	9	-	9	19	-	-
*A.baumanniiATCCBAA-47*	-	6,5	-	6,5	-	6,5	-	6,5	22	-	-
*En.aerogenesATCC 13048*	-	8	-	7	-	8	-	9	17	-	-
*Cb. freundii ATCC 43864*	-	7	-	7	6,5	7	6,5	7	20	-	-
*C. neteri ATCC 33855*	-	9	-	9	-	8	-	8	18	-	-
*Sa.typhimuriumATCC1402*8	-	7	-	7	-	-	-	-	-	-	-
*Pr. mirabilis ATCC 43071*	-	-	-	-	-	8	-	7	19	-	-
*Ca. albicans ATCC 10231*	7	8	7	9	6,5	7	-	6,5	-	24	-

Ch: Chloroform, DCM: Dichloromethane, H: Hexane, MeOH: Methanol, A: 15 μL, B: 30 μL

Tob: Tobramycin, Nys: Nystatin, Va: Vancomycin

a mg/disk

**Table 4:** Dichloromethane extract of *C. crinita* viability rate comparison at different hours.

Doses	3h	4h	7h	9h	11h	17h	20h	24h	30h	32h
Control	100.00 a	100.00 a	100.0 a	100.0 a	100.0 a	100.00 a	100.00 a	100.00 a	100.0a	100.00
117	90.89 ab	90.00 ab	78.20 ab	63.79 b	56.06 ab	49.00 b	31.48 b	19.23 b	8.17 b	0.00
234	86.34 abc	78.3 abc	63.00 ab	53.45 bc	49.30 bc	41.30 bc	27.78 bc	13.46 bc	0.00 c	0.00
468	77.50 bcd	66.67 abcd	57.90 ab	50.00 bc	45.70 bc	37.50 bc	18.52 bcd	11.54 bcd	0.00 c	0.00
937	65.54 cde	58.33 bcd	57.90 ab	48.28 bc	43.80 bc	33.80 bcd	14.81 cde	9.62 bcd	0.00 c	0.00
1875	61.12 de	55.00 cd	46.10 ab	36.21 bc	32.80 bc	20.73 bcd	11.11 de	7.69 bcd	0.00 c	0.00
3750	55.27 e	38.33 de	22.30 ab	25.86 cd	16.40 bc	7.48 cd	7.41 de	3.846 cd	0.00 c	0.00
7500	16.79 f	16.67 e	6.78 b	3.45 d	0.00 c	0.00 d	0.00 e	0.00 d	0.00 c	0.00
15000	11.96 f	10.00 e	1.72 b	0.00 d	0.00 c	0.00 d	0.00 e	0.00 d	0.00 c	0.00
**p**	**<0.001**	**<0.001**	**0.011** **	**<0.001**	**0.001**	**<0.001**	**<0.001**	**<0.001**	**<0.001**	**-**

-: not calculated

Means that do not share a letter are significantly different (p < 0.05).

** <0.01

**Table 5:** Hexane extract of *C. crinita* viability rate comparison at different hours.

Doses	3h	4h	7h	9h	11h	17h	20h	24h	30h	32h
Control	100.00 a	100.00 a	100.00 a	100.00 a	100.00 a	100.00 a	100.00 a	100.00 a	100.0a	100.0 a
117	82.05 b	78.33 ab	59.43 b	55.17 b	54.80 b	50.93 b	48.15 b	48.08b	48.92b	42.92 b
234	77.63 bc	78.33 ab	57.70 bc	48.28 bc	49.30 bc	47.15 b	44.44b	44.23b	42.83bc	40.75 b
468	68.79 c	75.00 b	55.98 bc	44.83 bcd	45.63 bc	41.45 b	40.74 b	42.31b	40.83c	38.75 b
937	58.26 d	63.33 bc	42.47 bc	43.10 bcd	45.57 bc	39.67 b	38.89b	38.46bc	38.75c	32.75 bc
1875	52.14 de	60.00 bc	39.02 bcd	36.21 cd	40.01 bcd	35.90 b	25.93 c	26.92c	30.58d	22.50 c
3750	46.30 e	46.67 c	33.97 cd	27.59 de	31.00 bcd	15.24 c	5.56 d	3.65d	3.67e	0.41 d
7500	25.22 f	43.33 c	17.07 de	17.24 ef	9.13 cd	2.81 c	0.00 d	0.00d	0.00e	0.00 d
15000	10.40 g	4.17 d	1.53 e	1.21 f	0.00 d	0.00 c	0.00 d	0.00 d	0.00 e	0.00 d
**p**	**<0.001**	**<0.001**	**<0.001**	**<0.001**	**<0.001**	**<0.001**	**<0.001**	**<0.001**	**<0.001**	**<0.001**

Means that do not share a letter are significantly different (p < 0.05)

**Table 6:** Chloroform extract of *C. crinita* viability rate comparison at different hours.

Doses	3h	4h	7h	9h	11h	17h	20h	24h	30h	32h
Control	100.0 a	100.00 a	100.00 a	100.0 a	100.00 a	100.00 a	100.00 a	100.00 a	100.0 a	100.0 a
117	56.83 b	58.33 b	59.37 b	55.17 b	52.78 b	45.37 b	42.59 b	32.69 b	30.58 b	20.50 b
234	55.27 b	53.33 bc	54.20 bc	53.45 b	50.79 bc	43.45 bc	35.19 bc	30.77bc	26.42 bc	16.42 b
468	52.28 b	50.00 bcd	50.92 bc	50.00 bc	40.08 bcd	37.75 bcd	29.63 cde	26.92 bc bc	18.33 cd	10.25 bc
937	47.99 b	43.33 bcde	44.14 bc	43.10 cd	38.23 bcd	35.90 bcd	31.48 cd	19.23 bcd	8.167 de	0.00 c
1875	46.43 b	41.67 bcde	42.30 bcd	43.10 cd	32.74 cde	32.12 cd	24.07 de	15.38 bcd	6.08 e	0.00 c
3750	39.02 b	38.33 cde	38.91 bcd	36.21 de	27.18 de	28.28 d	20.37 e	11.54 cd	0.00 e	0.00 c
7500	34.60 b	35.00 de	35.63 cd	32.76 e	14.62 ef	0.00 e	0.00 f	0.00 d	0.00 e	0.00 c
15000	33.17 b	31.67 e	20.46 d	5.17 f	0.00 f	0.00 e	0.00 f	0.00 d	0.00 e	0.00 c
**p**	**<0.001**	**<0.001**	**<0.001**	**<0.001**	**<0.001**	**<0.001**	**<0.001**	**<0.001**	**<0.001**	**<0.001**

Means that do not share a letter are significantly different (p < 0.05)

**Table 7:** Methanol extract of *C. crinita* viability rate comparison at different hours.

Doses	3h	4h	7h	9h	11h	17h	20h	24h	30h	32h
Control	100.00 a	100.0 a	100.00 a	100.00 a	100.00 a	100.00 s	100.0 a	100.0 a	100.0 a	100.00
117	47.72 b	45.00 b	30.57 b	27.59 b	27.31 b	24.50 b	24.07 b	13.46 b	6.08 b	0.00
234	46.30 b	36.67 bc	27.18 bc	20.69 bc	18.19 c	16.88 b	11.11 c	3.85 c	0.00 c	0.00
468	37.05 bc	35.00 bc	25.40 bc	15.52 bcd	12.70 c	5.63 c	2.778 d	0.00 d	0.00 c	0.00
937	26.52 cd	25.00 cd	18.62 bcd	12.07 cde	5.49 d	2.81 c	0.00 d	0.00 d	0.00 c	0.00
1875	17.95 cde	18.33 cde	11.95 cde	5.17 de	0.00 d	0.00 c	0.00 d	0.00 d	0.00 c	0.00
3750	16.52 de	18.33 cde	6.01 de	0.00 e	0.00 d	0.00 c	0.00 d	0.00 d	0.00 c	0.00
7500	13.53 de	6.67 de	2.53 de	0.00 e	0.00 d	0.00 c	0.00 d	0.00 d	0.00 c	0.00
15000	4.42 e	1.50 e	0.00 e	0.00 e	0.00 d	0.00 c	0.00 d	0.00 d	0.00 c	0.00
**p**	**<0.001**	**<0.001**	**<0.001**	**<0.001**	**<0.001**	**<0.001**	**<0.001**	**<0.001**	**<0.001**	**-**

-: not calculated

Means that do not share a letter are significantly different (p<0.05).

**Table 8:** NaCl extract of *C. crinita* viability rate comparison at different hours.

Doses	3h	4h	7h	9h	11h	17h	20h	24h	30h	32h
Control	100.00 a	100.00 a	100.00 a	100.00 a	100.00 a	100.00 a	100.00 a	100.00 a	100.00	100.00
117	71.65 b	71.67 b	62.76 b	60.34 b	52.84 b	48.93 b	37.04 b	26.92 b	0.00	0.00
234	68.67 b	63.33 b	59.37 bc	53.45 b	49.21 b	43.23 b	31.48 bc	25.00 bc	0.00	0.00
468	67.10 b	58.33 bc	52.59 bc	46.55 b	40.15 bc	35.75 bc	25.93 bc	19.23 bcd	0.00	0.00
937	49.15 c	53.33 bc	47.47 bc	37.90 bc	34.72 bc	31.98 bcd	22.22 cd	17.31 bcd	0.00	0.00
1875	44.73 cd	40.00 cd	37.5 cd	31.0 bc	23.81 bcd	16.95 cde	12.96 de	9.62 bcd	0.00	0.00
3750	38.75 de	30.00 de	23.79 de	15.52 cd	12.76 cd	9.40 de	7.407 ef	5.77 cd	0.00	0.00
7500	31.21 e	25.00 de	22.07 de	0.00 d	0.00 d	0.00 e	0.00 f	0.00 d	0.00	0.00
15000	16.38 f	11.67 e	5.11 e	0.00 d	0.00 d	0.00 e	0.00 f	0.00 d	0.00	0.00
**p**	**<0.001**	**<0.001**	**<0.001**	**<0.001**	**<0.001**	**<0.001**	**<0.001**	**<0.001**	**-**	**-**

-: not calculated

Means that do not share a letter are significantly different (p < 0.05).

**Table 9:** LD_50_ and LD_90_ values of *C. crinita* DCM-H extracts per time.

Time		Dichloromethane			Hexane		
		Value	95% Confidence Limit			95% Confidence Limit	
			Lower	Upper	Value	Lower	Upper
**3 h**	LD_50_	2649.4 a	2592.3	2707.4	1863.2 a	1813.1	1914.3
	LD_90_	16757.2 a	16187.3	17362.8	21649.4 a	20631.4	22752.9
**4 h**	LD_50_	1755.9 a	1711.5	1801.0	2401.6 a	2332.1	2473.0
	LD_90_	14567.7 a	14014.0	15159.9	28833.5 a	27200.5	30631.6
**7 h**	LD_50_	874.1 a	849.9	898.5	544.9 a	521.5	568.6
	LD_90_	7657.7 a	7394.2	7938.0	13948.2 a	13153.8	14825.0
**9 h**	LD_50_	498.5 a	480.4	516.8	321.0 a	302.9	339.4
	LD_90_	6800.0 a	6525.8	7094.9	13675.4 a	12786.2	14670.6
**11 h**	LD_50_	342.0 a	327.9	356.1	368.2 a	349.5	387.1
	LD_90_	4811.2 a	4621.3	5015.1	10679.5 a	10063.5	11361.4
**17 h**	LD_50_	185.7 a	175.0	196.7	289.8 a	274.3	305.5
	LD_90_	5062.6 a	4829.6	5315.3	8511.8 a	8056.5	9012.1
**20 h**	LD_50_	30.3 a	27.0	33.8	221.9 a	211.5	232.5
	LD_90_	1464.4 a	1399.3	1534.3	3258.6 a	3133.9	3392.3
**24 h**	LD_50_	3.5 a	2.8	4.5	227.5 a	216.8	238.2
	LD_90_	594.2 a	558.8	631.2	3150.2 a	3029.4	3279.7

a μg/mL

**Table 10:** LD_50_ and LD_90_ values of *C. crinita* Ch-MeOH extract per time.

Time		Chloroform			Methanol		
		Value	95% Confidence Limit		Value	95% Confidence Limit	
			Lower	Upper		Lower	Upper
**3 h**	LD_50_	601.0 a	546.9	657.1	117.9 a	109.4	126.8
	LD_90_	1542606.0 a	1108426.0	2219675.0	7693.1 a	7264.5	8165.2
**4 h**	LD_50_	441.9 a	396.0	489.8	90.8 a	83.5	98.3
	LD_90_	1278744.0 a	910975.0	1861004.0	5093.3 a	4826.0	5386.5
**7 h**	LD_50_	543.2 a	502.8	584.7	32.0 a	28.6	35.6
	LD_90_	187731.0 a	155686.0	229673.0	1863.2 a	1779.1	1953.8
**9 h**	LD_50_	445.6 a	417.1	474.8	27.6 a	24.8	30.6
	LD_90_	48115.4 a	42865.6	54390.6	738.1 a	709.8	767.9
**11 h**	LD_50_	270.4 a	254.5	286.6	35.9 a	32.6	39.4
	LD_90_	10436.6 a	9804.9	11138.9	460.5 a	444.6	477.1
**17 h**	LD_50_	203.2 a	189.3	217.4	2.7 a	2.1	3.5
	LD_90_	10670.7 a	9967.8	11459.7	856.5 a	804.4	911.8
**20 h**	LD_50_	101.1 a	93.2	109.3	44.1 a	40.4	47.7
	LD_90_	4316.4 a	4094.3	4560.0	239.1 a	232.2	246.2
**24 h**	LD_50_	44.9 a	40.3	49.7	33.1 a	29.0	37.1
	LD_90_	2299.7 a	2188.5	2420.4	142.1 a	137.3	146.8

a μg/mL

**Table 11:** LD_50_ and LD_90_ values of NaCl per time.

Time		NaCl		
		Value	95% confidence limit	
			Lower	Upper
**3 h**	LD_50_	1305.8^a^	1258.1	1354.6
	LD_90_	40220.2 a	37256.1	43563.4
**4 h**	LD_50_	907.8 a	872.9	943.4
	LD_90_	23128.4 a	21672.9	24746.5
**7 h**	LD_50_	558.6 a	534.3	583.2
	LD_90_	15365.0	14468.1	16356.5
**9 h**	LD_50_	370.0 a	356.4	383.7
	LD_90_	4331.7	4177.7	4495.9
**11 h**	LD_50_	245.6 a	234.5	256.8
	LD_90_	3593.4	3457.3	3739.0
**17 h**	LD_50_	176.8 a	166.4	187.4
	LD_90_	4885.6 a	4662.5	5127.3
**20 h**	LD_50_	63.6 a	58.4	69.0
	LD_90_	1971.1 a	1888.1	2060.3
**24 h**	LD_50_	23.2 a	20.3	26.3
	LD_90_	1327.9 a	1266.0	1394.3

a μg/mL
